# Potential angiogenic, immunomodulatory, and antifibrotic effects of mesenchymal stem cell-derived extracellular vesicles in systemic sclerosis

**DOI:** 10.3389/fimmu.2023.1125257

**Published:** 2023-05-12

**Authors:** Kelin Zhao, Chenfei Kong, Naixu Shi, Jinlan Jiang, Ping Li

**Affiliations:** ^1^ Department of Rheumatology and Immunology, China-Japan Union Hospital, Jilin University, Changchun, China; ^2^ Scientific Research Center, China-Japan Union Hospital, Jilin University, Changchun, China; ^3^ Department of Stomatology, China-Japan Union Hospital, Jilin University, Changchun, China

**Keywords:** systemic sclerosis, fibrosis, mesenchymal stem cell, extracellular vesicle, vascular injury

## Abstract

Systemic sclerosis (SSc) is an intricate systemic autoimmune disease with pathological features such as vascular injury, immune dysregulation, and extensive fibrosis of the skin and multiple organs. Treatment options are limited; however, recently, mesenchymal stem cell-derived extracellular vesicles (MSC-EVs) have been acknowledged in preclinical and clinical trials as being useful in treating autoimmune diseases and are likely superior to MSCs alone. Recent research has also shown that MSC-EVs can ameliorate SSc and the pathological changes in vasculopathy, immune dysfunction, and fibrosis. This review summarizes the therapeutic effects of MSC-EVs on SSc and the mechanisms that have been discovered to provide a theoretical basis for future studies on the role of MSC-EVs in treating SSc.

## 1 Introduction

Systemic sclerosis (SSc) is a complex and chronic connective tissue disorder with an incidence of 17.6 per 10,000 (1). The pathogenesis of SSc is dominated by disorders in three major areas of pathophysiology: vasculopathy, immune dysfunction, and fibrosis. The disease progression is cumulative with amplified effects that primarily involve endothelial cell (EC) activation and the recruitment of inflammatory cells, followed by the release of various factors. This results in fibroblast activation and the deposition of extracellular matrix (ECM) proteins (2).

However, the etiology of SSc is complex and unclear since epigenetics, environmental factors, and a history of infection and drugs may all contribute to SSc. Genetics is the primary factor, and a survey has shown the percentage of relatives of patients with SSc suffering from the disease is higher than that of the general population (3). In addition, genetic factor studies at different levels of genetic information have advanced considerably with the development of technology and lower costs. There is a close relationship between human leukocyte antigen (HLA) locus genes and SSc, and 32 non-HLA loci have been identified (4). This opens up promising possibilities for the use of precision medicine for SSc patients. It has been shown that viruses could trigger SSc, in particular parvovirus B19 (5), cytomegalovirus, Epstein–Barr virus, and retroviruses (6). Intriguingly, recent findings that SARS-CoV-2 may be involved in the occurrence of SSc have been disputed. Although COVID-19 in patients only affects the incidence of SSc minimally (7), both diseases have manifestations of endothelial damage, making it possible for them to correlate (8).

Clinically, SSc is primarily characterized by skin fibrosis and the accelerated progression of organ-associated complications, which include the early appearance of arthropathy, gastrointestinal dysmotility, myositis, and Raynaud’s phenomenon (9), and late stages of severe pulmonary or cardiac complications with significant morbidity and mortality rates (10). Further characteristics of SSc include a prevalence rate that is approximately four times higher in women than that in men, an age of onset between 30 and 60 years (1), an incidence rate higher in North America than that in northern Europe (11), and a more rapid and severe disease progression in African-American patients (12).

Faced with cumulative and persistent multi-organ symptoms, no single approach to the treatment of SSc has proven uniformly effective. Current clinical treatments are mainly palliative, and current management strategies focus on treating the symptoms, accompanied by systemic immunotherapy (13). Early intervention with drugs for vascular modulation, especially during the early symptoms of vascular injury, substantially mitigates sclerosis associated with pulmonary hypertension and reduces the risk of mortality associated with scleroderma renal crisis (14, 15). Immunosuppressive drugs are typically used during the active and diffuse phases of the disease and may result in a poor prognosis for patients with SSc (13). In addition, hematopoietic stem cell transplantation and several agents targeting potential drivers of disease pathogenesis, such as T cells, B cells, transforming germinal factor (TGF) β, and interleukin (IL)-6, are under evaluation as possible therapeutic agents in clinical trials (16–18).

Mesenchymal stem cells (MSCs) have the capacity to not only modulate immune cell activity but also stimulate tissue regeneration, mainly by secreting extracellular vesicles (EVs), which in turn play a role in the functional treatment of diseases (19). EVs secreted by MSCs contain a variety of bioactive substances such as DNA, mRNA, long non-coding RNA (lncRNA), proteins, and lipids and may be classified into three subtypes based on their size: exosomes (Exos; 30–100 nm), microvesicles (50–2,000 nm), and apoptotic bodies (50–5,000 nm) (20). Exos have the highest degree of homogeneity and are the most complex and versatile of the three types. Therefore, due to the highest value of theory and application, Exos receive the most attention in academic papers on EV. MSC-EVs can be classified as adipose tissue-derived MSCs (ASCs), bone marrow MSCs (BM-MSCs), umbilical cord MSCs (UC-MSCs), menstrual fluid MSCs (MenSCs), human-induced pluripotent stem cell MSCs, and human amniotic fluid MSCs (AF-MSCs) (21). EVs from ASCs, UC-MSCs, and BM-MSCs exhibit a capacity for wound healing; however, BM-MSC-EVs have stronger induction effects on fibroblasts, and the greatest induction of keratinocytes belongs to UC-MSC-EVs (22). Thus, when considering their use, it should be noted that EVs from different sources of MSCs can differ in efficacy.

There are still some challenges in the clinical application of MSC-EVs, for instance, high and sustained production, prolonged *in vivo* action, and avoidance of macrophage clearance (23, 24). However, compared to MSC transplantation, MSC-EVs possess a number of advantageous characteristics, including smaller size, singularity, long circulatory half-life, low immunogenicity, easy coating of therapeutic substances, easy crossing of the blood–brain barrier, easy production and storage, and no tumorigenicity (25). Therefore, various clinical studies are currently underway regarding the therapeutic applications of MSC-derived EVs (MSC-EVs) in autoimmune diseases (ADs) (26), including SSc. This review summarizes the possible pathogenesis of SSc and explores the potential use of MSC-EVs in SSc treatment. MSC-EVs may contribute to the treatment of SSc by improving vascular lesions, regulating immune dysfunction, and inhibiting fibrosis.

## 2 Pathophysiology of systemic sclerosis

Despite the fact that the exact causes of SSc are not well understood, numerous studies have shown that endogenous and exogenous environmental factors or risk factors trigger gene activation. The subsequent onset of SSc is associated with endothelial damage, microvascular injury, inflammation, and autoimmune activation (27, 28). These factors inevitably cause abnormal differentiation of fibroblasts and the accumulation of collagen and ECM proteins in tissues. SSc progression is discussed below in three parts: vascular injury, the immune response, and fibrosis.

### 2.1 Vascular injury and microangiopathy

EC activation is the main event at the beginning of SSc (29), wherein the enhanced expression of adhesion molecules, such as vascular cell adhesion protein 1, intercellular adhesion molecule, and E-selectin, lead to activation of the abnormal secretion of vasoactive factors (30). The adhesion molecules with adhesion mainly recruit inflammatory cells, while the disturbed vasoactive factors lead to frequent and constant fluctuations in microvascular tone (31). Platelet activation, which is caused by vascular changes, increases microvessel permeability; therefore, microvascular leaks can develop (32). Moreover, platelet activation enhances the proliferation of vascular smooth muscle cells (VSMCs) and pericytes, leading to a thickening of the vessel wall and luminal narrowing (31). These events then cause microvascular damage, tissue hypoxia, and oxidative stress (33).

### 2.2 Inflammation and the immune response

In the early inflammatory phase of SSc, Toll-like receptor (TLR) signaling, which acts as a significant indicator of inflammation, can be provoked by non-specific or pathogenic injury, which may result in inflammation induction and the activation of innate immune cells (monocytes/macrophages, plasmacytoid dendritic cells (pDCs), and others). The upregulated production of CXCL4 in plasmacytoid DCs can lead to the differentiation of monocytes into pro-inflammatory DCs that enhance TLR-mediated cytokine expression and impact T cells (34, 35). In addition, monocytes participate in fibrosis through the inflammatory response and differentiate into macrophages or fibroblast-like cells. Macrophages are also engaged in the inflammatory and fibrotic aspects of SSc. Macrophages can generate classically activated (M1) and/or alternatively activated (M2) macrophages that are distinguishable based on their different surface markers. M1 macrophages are effector phagocytes that increase significantly in the early stage of SSc inflammation and generate pro-inflammatory cytokines, such as tumor necrosis factor-alpha (TNF-α), IL-6, and IL-1 (36). M2 macrophages restrain M1 responses by releasing anti-inflammatory cytokines, including IL-4, IL-13, and IL-10 when the repair mechanism is initiated after sustained damage. In addition, they facilitate the production of ECM proteins and pro-fibrotic cytokines and reinforce the anti-inflammatory response by triggering Th2 effector activity (36). Thus, M2 macrophages are considered to be important pathogenic factors in SSc.

Among the various adaptive immune responses, T cells participate significantly in the pathophysiology of SSC and markedly affect the synthesis of autoantibodies (37). CD4+ and CD8+ T cells have been confirmed in the skin (38, 39) and lungs (40, 41) of patients with SSc. Initial CD4+ T cells (Th0) can differentiate into Th1, Th2, and Th17, as well as Treg (T-regulatory) and Tfh (T-follicular helper) cells (42–44). Th2 cells, characterized by the secretion of anti-inflammatory cytokines IL-4 and IL-13, predominate over Th1 cells (45). Moreover, both Th17 cells and IL-17 production have been recognized as being elevated in patients with SSc, and they aggravate early inflammatory responses (46, 47). Treg cells could result in the advancement of SSc by transforming into pathogenic effector T cells, and their number can be reduced (48, 49) to inhibit immune activation (50). Therefore, an imbalance of Th1/Th2/Th17/Treg cytokines is a crucial causal factor for SSc. B cells also play a role in SSc, and their efficacy is linked to antigen presentation, DC maturation, and autoantibody production, varying across phenotypes (51). Different phenotypes exhibit different CD antigens on the cell surface, such as memory B cells with increased expression of CD95, CD80, and CD86, and peripheral blood B cells with a higher expression of CD19 (52–55).

### 2.3 Fibrosis

Due to persistent tissue damage, inflammation, and immune cell activation, SSc comprises a gradual fibrotic condition affecting tissues and organs (56). Clinical and pathological SSc is characterized by fibrosis accompanied by massive α-SMA-positive myofibroblasts, accumulation of ECM proteins (collagens, elastin, glycosaminoglycans, tenascin, and fibronectin) in tissue, and regulation of growth factor (TGF)-β and other profibrotic mediators (57). Circulating CD14 monocyte precursor pericytes and ECs have been suggested as potential sources of myofibroblast overproduction through epithelial–mesenchymal transition and endothelial–mesenchymal transition (58). The subsequent steady accumulation of ECM proteins stiffens the skin and organs while decreasing elasticity, thereby leading to mechanical stress. Mechanical stress further maintains fibroblast activation and intensifies the progression of the fibrotic process in tissues (59). This may be due to fibroblasts in patients with SSc exhibiting structural focal adhesion kinase activation, which integrates TGF-β signaling and integrin-mediated mechanical transduction so as to promote continuous myofibroblast differentiation and reactive oxygen species production (59).

The soluble mediators related to fibrosis in SSc are TGF-β, connective tissue growth factor (CTGF), and platelet-derived growth factor (PDGF). TGF-β, a pleiotropic factor secreted by macrophages and other cells or stored in the ECM, is believed to be the master regulator of fibrosis. As an inactive precursor, TGF-β-associated signaling cascades are consistently activated in fibrotic tissues (60). CTGF, a cysteine-rich matricellular protein, has a synergistic effect together with TGF-β, endothelin-1, and angiotensin II in inducing fibrosis (61). CTGF levels are significantly higher in the sera of SSc patients than those in the sera of healthy individuals and are positively correlated with the degree of fibrosis (62). PDGFs, which are heterodimeric peptides produced by platelets, macrophages, ECs, and fibroblasts, play an important role in fibrosis as well. PDGFs act as powerful mitogens and chemoattractants and convert mesenchymal cells into profibrotic cell types (63).

## 3 Potential therapeutic effects of MSC-EVs in SSc

**Figure 1:**
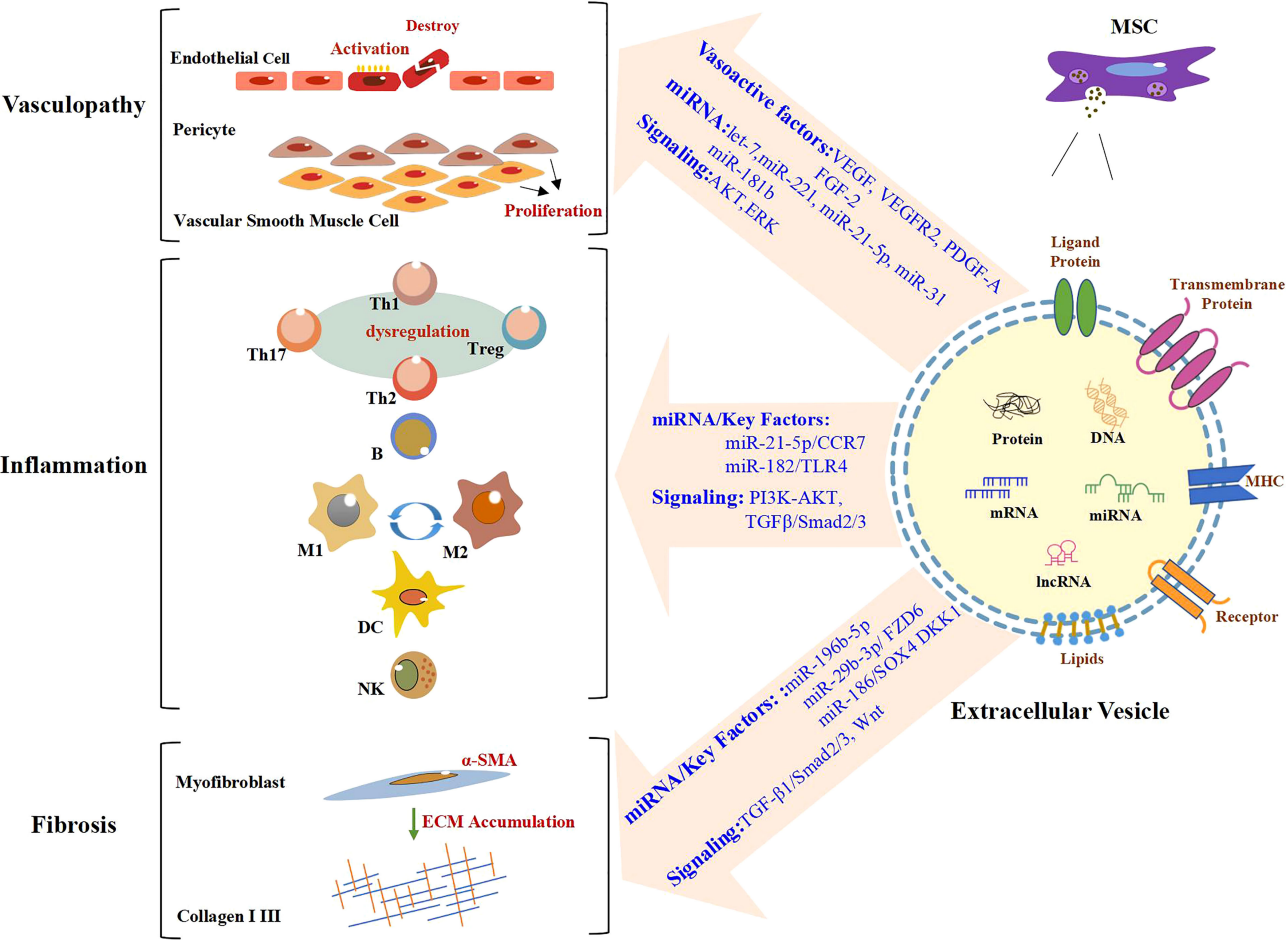
Role of MSC-EVs in the modulation of SSc. In the stage of vasculopathy in SSc, endothelial cells are activated and even destroyed. Vascular smooth muscle cells and pericytes proliferate abnormally. A large number of immune cells also participate in the process of SSc. Likewise, there are large numbers of α-SMA-positive myofibroblasts, which produce excessive amounts of extracellular matrix (ECM), in particular Collagen I and III, resulting in fibrosis. MSC-EVs promote angiogenesis by regulating vasoactive factors and signaling pathways and carrying miRNA. MiRNA/key factors and signaling pathways are involved in the modulation of inflammation and fibrosis by MSC-EVs. Text in blue indicates mechanisms involved in SSc. MSC-EVs, mesenchymal stem cell-derived extracellular vesicles; SSc, systemic sclerosis.

MSC-EV transplantation, which is an emerging and novel therapy, has been confirmed to be beneficial for SSc in bleomycin or hypochlorous acid (HOCl)-induced, or chronic graft-versus-host disease (cGVHD) mouse models, and TGF-β1-induced model of human myofibroblast (**Table 1**). These preclinical studies are mainly focused on the antifibrotic effect of MSC-EVs in SSc and the mechanisms involved (64, 65). The discovery of effective improvements of MSC-EVs in SSc *in vitro* and *in vivo* is a breakthrough in the field of SSc therapeutic approaches. Recent advances have been made in the exploration of the biogenesis, cargo, and biological potential of MSC-EVs and in understanding their molecular mechanisms in angiogenesis and immunomodulation, which is the key to SSc treatment. These advances have led to promising improvements being observed in other diseases through MSC-EV treatment (71, 72). In view of the heterogeneity of pathways involved in SSc pathogenesis and progression, as well as the existence of crosstalk among vascular injury, the immune response, and fibrosis in SSc, long-term single-target treatment could lead to adverse reactions (73, 74). Thus, for SSc, MSC-EVs enriched with multiple efficacious biokines perhaps improve through various pathways, which may include angiogenesis and the modulation of inflammation and fibrosis (**Figure 1**). This evidence points out that MSC-EVs may become a potential tool for SSc. The effects of MSC-EVs on vascular injury, immune imbalance, and fibrosis in different disease models are described below.

**Table 1:** Effect of MSC-EVs on SSc.

	Source of MSCs	Experiment models	Properties of MSC-EVs	Mechanisms	References
1	Human umbilical cord	Bleomycin-induced mouse model	Antifibrosis	Reduction of deposition of extracellular matrix and inhibition of the epithelial–mesenchymal transition process	(64)
			Immunomodulation	Facilitating M1 macrophage polarization and suppressing M2 macrophage polarization, leading to the restoration of the balance of M1/M2 macrophages	
2	Adipose tissue	TGF-β1-induced model of human myofibroblasts	Antifibrosis	ASC-EVs can better improve anti-fibrotic and pro-remodeling functions than ASCs *in vitro*	(65)
3	Bone marrow	HOCl-induced murine model	Antifibrosis	IFN-γ-pre-activation improved the therapeutic effects of MSC-EV in the SSc model. Low doses of IFN-γ decreased the expression of fibrotic markers, while high doses improved remodeling markers *in vivo*	(66)
			Immunomodulation	High dose of IFN-γ-pre-activation upregulated anti-inflammatory markers in MSC-EV, including iNos, IL1ra, and IL-6 *in vivo*	
4	Bone marrow	Bleomycin-induced dermal fibrosis in mice	Antifibrosis	Inhibition of collagen type I expression by miR-196b-5p in exosomes might be one of the mechanisms by which MSCs suppress skin fibrosis *in vivo*	(67)
5	Bone marrow	Bleomycin-induced dermal fibrosis in mice	Antifibrosis	The intervention of fibrosis of the SSC model by miRNAs they carry and regulate the WNT and TGF-β signaling pathways	(68)
			Immunomodulation	Reduction of the numbers of mast cells and infiltrating macrophages and lymphocytes, and the levels of the inflammatory cytokines IL-6, IL-10, and Tnf-α in BLM-treated mice	
6	Human umbilical cord	Bleomycin-induced dermal fibrosis in mice	Antifibrosis	Attenuating myofibroblast activation and collagen deposition in dermal fibrosis by downregulating the TGF-β/Smad signaling pathway *in vivo*	(64)
7	Human adipose tissue	HOCl-induced SSc model	Antifibrosis	Alleviating SSc and regulating methylation and apoptosis *via* miR-29a-3p	(69)
8	Human umbilical cord	cGVHD mouse model	Immunomodulation	Suppressing the activation of macrophages and B-cell immune response	(70)

MSC-EVs, mesenchymal stem cell-derived extracellular vesicles; SSc, systemic sclerosis; MSCs, mesenchymal stem cells; ASCs, adipose tissue-derived mesenchymal stem cells; HOCl, hypochlorous acid; BLM, bleomycin; cGVHD, chronic graft-versus-host disease.

### 3.1 Pro-angiogenic effects

Vascular injury and the thickening of the vessel wall are key components of SSc. In the early stage of SSc, if the extent of the vascular injury can be reduced, subsequent fibrosis can be considerably inhibited (75). Recent studies have demonstrated the MSC-EV-mediated delivery of cytokines, proteins, microRNA (miRNA), mRNA, and lncRNA as a significant component of the angiogenic process (72, 76). MSC-EVs have been shown to have pro-angiogenic properties in wound-healing models and ischemic conditions such as diabetic foot ulcers (DFUs), full-thickness wounds, myocardial infarction (MI), and acute kidney injury.

#### 3.1.1 Angiogenic function of MSC-EVs

ECs boost angiogenesis (the formation of fresh blood vessels from existing ones) and the secretion of several factors, including nitric oxide, endothelin, and prostacyclin (77). Therefore, they are valuable targets under ischemic conditions and require vascular regeneration. Studies have indicated that MSC-EVs enhance the migration of human umbilical vein endothelial cells (HUVECs) and promote proliferation and vessel-like structure formation in a dose-dependent manner in human microvascular endothelial cells (HMECs) *in vivo* and *in vitro* (78, 79). Another research has shown that MSC-Exos are internalized by HUVECs and accumulate around the nucleus (80). The characteristics of MSC-EVs can influence pathological changes in vascular-rich tissue, similar to Raynaud’s phenomenon, which affects over 95% of SSc patients and comprises occasional color changes in the extremities in cold environments (81). SSc-associated interstitial lung disease (SSc-ILD), one of the causes of death, is chiefly caused by damage to the alveolar epithelium and blood vessels in the lung region (82). Similarly, MSC-EVs are effective against other structural cells that are associated with blood vessels. Studies on abnormal proliferation and the migration of smooth muscle cells have demonstrated that MSC-EVs can alleviate asthma or pulmonary arterial hypertension *in vivo* and/or *in vitro* (83, 84). On this basis, MSC-EVs are promising as prospective therapeutic targets for SSc-related vasculopathy.

#### 3.1.2 Angiogenic molecular mechanisms of MSC-EVs

MSC-EVs most likely promote angiogenesis by upregulating vasoactive factors such as vascular endothelial-derived growth factor (VEGF), VEGF receptor (R)2, PDGF-A, and fibroblast growth factor (FGF)-2 in HUVECs *in vitro* and *in vivo* skin injury models (85). Studies conducted on primary MSCs originating from BM, ASCs, and UCs have revealed that all of the aforementioned cell-derived Exos promote angiogenesis by secreting angiogenesis-mediated factors such as VEGF-A, FGF-2, hepatocyte growth factor, and PDGF-BB during wound healing (22). In addition, exposure to vasoactive factors can strengthen the therapeutic effect of MSC-EVs and encourage better angiogenesis. Lopatina et al. previously showed that PDGF pretreatment of human ASC-EVs reinforces pro-angiogenic capacity by elevating the levels of secreted pro-angiogenic proteins. Conditioned media derived from AF-MSCs incorporating VEGF and TGF-β1 have been found to improve proliferation, the migration of human skin fibroblasts *in vitro*, and wound healing *in vivo* (86). As for the angiogenic effects of MSC-EVs, vasoactive factors may be targeted by MSC-EVs, which would influence the properties of MSC-EVs.

Other angiogenic mechanisms that warrant further study are the signaling pathways that involve MSC-derived proteins. A comprehensive analysis previously showed that ASC-EVs contain proteins that are linked to signal transduction pathways. A total of 277 proteins associated with the ECM, glycoproteins, angiogenesis, TGF-β signaling, the inflammatory response, and blood coagulation have been found to be enriched in EVs. It has been found that ASC-EVs facilitate angiogenesis through increased expression of Ang-1 and Flk-1 in HUVECs, involving the let-7/argonaut 1/VEGF signaling pathway in a fat grafting nude mouse model (87). Additionally, ASC-MVs have been found to be readily internalized by HUVECs. ASC-MV proliferation, migration, and angiogenesis are promoted through the AKT and ERK signaling pathways *in vitro* and in an *in vivo* skin injury model (85). The activation of these signaling pathways often involves not only proteins but also genetic materials such as miRNA, mRNA, and lncRNA.

It has been found that although MSC-EVs carry different genes than their parent cells, they can still regulate angiogenesis. Eirin et al. found that 386 miRNAs were enriched in ASC-EVs compared to parental cells. MicroRNA-148, one of these detected miRNAs, regulates angiogenesis aimed at transcription factors, inhibits tumor angiogenesis, and suppresses the sprouting of ECs from vessels *in vivo* and/or *in vitro* (88–90). Other miRNAs and even genes in their families also act independently from the key factors related to regulatory angiogenesis (91–94). Additionally, miR-221 was found to impact vasoactivity by exerting effects on VSMCs and ECs (95). Meanwhile, EVs obtained from MSCs have been known to exhibit a high level of pro-angiogenic miRNA-21-5p. The pro-angiogenic function of miRNA-21-5p in MSC-EVs was verified in DFU models by knockdown and overexpression of the gene, and it was also further determined that miRNA-21-5p may promote angiogenesis and improve ischemic tissue by stimulating VEGFR and by activating serine/threonine kinase AKT and mitogen-activated protein kinase in receptor cells such as ECs (96). Furthermore, for ischemic diseases, studies revealed that miR-31 was rich in ASC-MVs, especially in endothelial differentiation medium-pretreated ASC-MVs, and that miR-31 has been strongly implicated in angiogenesis in HUVECs and endothelial progenitor cells (97) in promoting migration and tube formation *in vitro* and *in vivo*. Moreover, both the miR-31-miR-720 and the VEGF-miR-31 pathways may be involved (98, 99). In addition, factor-inhibiting HIF-1, an anti-angiogenic gene, has been identified as a target of miR-31 in HUVECs, and together they may mediate angiogenesis (79). Additional studies have shown that MSC-Exos and its Exo-transmitted miR-125a can promote endothelial tip cell generation for EC, resulting in vascular sprouting by inhibiting the expression of DLL4, an angiogenesis inhibitor *in vivo* and *in vitro* (100). ASC-Exos promote the mobility and angiogenesis of brain microvascular endothelial cells after oxygen-glucose deprivation *via* the microRNA-181b/TRPM7 axis *in vitro* (101). Significant progress has been made in the study of miRNA in MSC-EVs. These miRNAs are not only involved in MSC-EVs promoting angiogenesis but also have immunomodulatory and antifibrosis effects (**Table 2**). MSC-EV delivery can protect miRNAs from degradation by ribonucleases, thereby ensuring that the miRNAs are able to perform their crucial roles in recipient cells. Interestingly, another study showed that proteins in EVs do not originate from the transcription of miRNAs.

**Table 2:** The miRNA of MSC-EVs is involved in angiogenesis, immunomodulation, and antifibrosis.

	Properties of MSC-EVs	Classification of MSC-EVs	MiRNA	References
1	Angiogenesis	Porcine ASC-EVs	MicroRNA-148	(88, 90)
		Human MSC-Exos	MiRNA-21-5p	(96)
		ASC-MVs	MiRNA-31	(79)
		MSC-Exos	MiRNA-125a	(100)
		ASC-Exos	MicroRNA-181b	(101)
2	Immunomodulation	MSC-Exos	MiRNA-23a-3p	(102)
		BM-MSC-Exos	MiRNA-146a-5p	(103)
		MSC-Exos	MiRNA-125a and miRNA-125b	(104)
		ASC-Exos	MiRNA-10a	(105)
		MSC-Exos	MiRNA-182	(106)
		BM-MSC-Exos	MiRNA-34c-5p	(107)
3	Antifibrosis	MSC-EVs	MiRNA-29a-3p	(69)
		MSC-EVs	MicroRNA-29b-3p	(108)
		BM-MSC-EVs	MicroRNA-186	(109)
		MSC-Exos	MiRNA-196b-5p	(67)

### 3.2 Immunomodulatory effects

Abnormal immune cells were exhibited in SSc focusing on the dysregulation of the Th1/Th2/Th17/Treg and M1/M2 cytokine (2). MSC-EVs have an auto-immunosuppressive property that attenuates inflammation and immune responses in inflammatory and autoimmune diseases (119). MSCs act as immunomodulators by delivering EVs that modulate the generation, differentiation, efficacy, and interactions of adaptive and innate immune cells, such as T cells, B cells, macrophages, natural killer (NK) cells, and DCs in inflammatory diseases (120). Likewise, the anti-inflammatory and immunomodulatory properties of MSC-EVs are probably of great significance in the treatment of SSc.

#### 3.2.1 T cells

In the early stages of SSc, the inhibition of inflammation is an important goal of therapy. At this stage, M1 macrophages and Th1 cells are the main cells involved in the upregulation of type I interferon (IFN) (121). In tissues, M1 macrophages with IFNs are transformed into M2 macrophages to promote fibrosis, after synergistic involvement with the responses of Th2 to exposure to IL-6 and IL-13 (122). Increased numbers of Treg cells facilitate the inhibition of immune activation and prevent themselves from transferring to Th2- and Th17-like cells for antifibrotic progress in skin tissues (123). As MSC-EVs activate different effector substances depending on different biological environments, the disorder in SSc could be regulated by them.

##### 3.2.1.1 Th cells

MSC-EVs modulate the functions and activities of Th cells. It has been demonstrated that MSC-EVs regulate CD4 T cells in the conversion between Th1 and Th2 and decrease the Th17 differentiation of peripheral blood mononuclear cells in asthmatic mice (124). In dextran sulfate sodium-induced colitis models, olfactory ecto-derived MSC-EVs have been found to remarkably reduce Th1/Th17 subpopulations, accompanied by reduced serum levels of IL-17, IL-6, and IFN-γ and elevated levels of TGF-β and IL-10 secreted by T cells (125). Interestingly, pro-inflammatory cytokines, such as IL-10 (126) and IFN preconditioned MSC, could enhance CD4 T-cell inhibition while inducing Treg cells and Th17. In addition, the molecular mechanisms of the MSC-EV-mediated regulation of Th cells focus on cargoes in EV, for example, by transferring miR-23a-3p to regulate the Treg/Th17 balance in aplastic anemia (102), miR-146a-5p/IRAK1 axis to regulate the Th17/Treg imbalance in immune thrombocytopenia (103), miR-125a and miR-125b to inhibit Th17 cell differentiation in colitis (104), and miR-10a loading to promote Th17 and Treg responses while decreasing Th1 responses (105).

##### 3.2.1.2 Treg cells

MSC-EVs substantially promote the conversion of monocytes to Treg cells and the immune-suppression capacity of Treg cells (127, 128), which is possibly triggered by TGF-β from MSC-EVs exposed to IFN-γ (129). Meanwhile, MSC-EVs could lead to the differentiation of Treg cells to reverse the imbalance between T-effector and Treg cells, which has been confirmed regarding the imbalance of Th1/Th4/Th17/Treg cells (105). It has been noted that Treg regulation occurs mainly through antigen-presenting cells (APCs) and is not dependent on CD4+ T cells in asthmatic mice (124). Subsequent studies have also confirmed that the differentiation of Treg cells is mediated by activated APCs, which are induced by MSC-EVs in a myeloid differentiation primary response-dependent manner (128). Treg differentiation can also suppress the proliferation of T cells indirectly, which is a property of MSC-Exos, but not of MSCs (130). The ability of MSC-EVs to regulate the function, homing, and phenotype of immune cells may result in the development of novel therapies for inflammatory diseases and ADs (26, 120). Nevertheless, further research is needed to elucidate the molecular mechanisms of the MSC-EV-mediated regulation of immune cell activities.

#### 3.2.2 B lymphocytes

B lymphocytes play a major role in the adaptive immune response and MSC-EVs have been shown to regulate the proliferation and function of B cells. Khare et al. (131) sequenced MSC-EV co-cultured with B cells and identified upregulated genes related to B-cell proliferation and Ca^2+^ mobilization *via* B-cell receptors *in vitro*. Guo et al. earlier revealed that MSC-EVs suppressed fibrosis in a mouse model of sclerodermatous chronic graft-versus-host disease by inhibiting Tfh/germinal center B-cell interactions and reducing the frequency of B cell-activating factor (BAFF)-expressing B cells (70). Furthermore, MSC-EVs have been found to regulate Breg cells through the PI3K-AKT signaling pathway *in vitro*, but the role of MSCs in regulating Breg cells still needs to be explored (132).

#### 3.2.3 Macrophages

Macrophages orchestrate both the initiation and resolution of inflammation. An imbalance in the phenotypes and activation of macrophages has been considered critical in the development of inflammatory, autoimmune, and fibrotic diseases. Accumulating evidence has suggested that MSC-EVs promote macrophage polarization and phagocytic capacity (133). The regulation of EVs in the conversion between M1 and M2 macrophages has also been found in some cases of SSc (134). Meanwhile, MSC-EVs reduce macrophage infiltration in tissues by carrying miRNAs in SSc (68). Interestingly, in renal interstitial fibrosis, Hu et al. (107) showed that MSC-Exos may inhibit macrophage activation by delivering miR-34c-5p in Exos to macrophages *via* CD81-epidermal growth factor receptor (EGFR) complex aids. In another context, it was noted that MSC-EVs could reverse the polarization of M1 to M2 macrophages; further, it was found that miR-182 from MSC-EVs can suppress TLR4, as part of its underlying mechanism of action in myocardial ischemia–reperfusion injury (106).

#### 3.2.4 DCs and NK cells

DCs and NK cells are essential for the innate immune response, and the effect of MSC-EVs on both cells has been explored in some studies *in vitro*. Several reports have shown that MSC-EVs could suppress the proliferation and maturation of DCs, thus regulating the immune response. The coincubation of DCs derived from BM with MSC-EVs has been found to reduce IL-6 release, increase IL-10 and TGF-β levels, and downregulate lymphocyte proliferation (135). MSC-EVs loaded with miR-21-5p are able to depress the target gene C–C chemokine receptor type 7 (CCR7) and reduce the migratory capacity of the CCR7-ligand CCL21, thereby suppressing the secretion of inflammatory cytokines (136). Previous studies have demonstrated that MSC-EVs could suppress the proliferation of NK cells in a manner similar to DCs (130). In recent research, an analogous study showed that human fetal liver-derived MSC-Exos not only inhibited NK-cell proliferation but also suppressed NK-cell activation and cytotoxicity *via* latency-associated peptides and TGF-β and thrombospondin 1 and induced downstream TGF-β/Smad2/3 signaling. Nevertheless, research on the topic remains limited. Despite the growing research on MSC-EVs and immune cells, the precise molecular mechanisms underlying their interactions needs additional study (137). Innate immune responses also occur in the course of SSc. Regulation of DCs and NK cells by MSC-EVs should play a role in SSc.

### 3.3 Antifibrotic effects

MSC-EV treatment can ameliorate fibrosis of the skin and lungs, which is a clear sign of SSc. Furthermore, the clinical fibrotic manifestations of SSc involve alterations in the heart, kidney, and colon, and several experimental studies have examined the antifibrotic effect of MSC-EVs in other diseases (**Table 3**). Presently, investigations of fibrosis are restricted to the preclinical phase. Many preclinical trials have been conducted to set up various SSc models. There are many methods to induce SSc given the various underlying causes of fibrosis (**Supplementary Table 1**); however, no experimental models have been able to perfectly reproduce the pathophysiological spectrum of SSc.

**Table 3:** The fibrotic target tissues of MSC-EVs.

	Target tissues	Target cells	Results	Key mechanisms	References
1	Skin	Fibroblasts	Promotion of proliferation and migration in SSc	MiR-29b-3p/PI3K-Akt, Erk1-2, and Smad3-TGF-β1	(110)
		Myofibroblasts	Suppressing myofibroblasts differentiation	MicroRNAs (miR-21, miR-23a, miR-125b, and miR-145)/TGF-β-SMAD2	(111)
2	Lung	Lung epithelial cells	Better proliferative capacity of alveolar epithelial cell line	Activation of FGF2 signaling	(112)
		Pulmonary vascular endothelial cells	Reduction of tissue fibrosis and vascular endothelial remodeling	Umbilical cord MSC-exosomal TNF-stimulated gene 6	(113)
		Alveolar macrophages	Alleviating lung inflammation and fibrosis	Polarization of macrophages to m2 anti-inflammatory phenotype	(114)
3	Heart	Cardiomyocytes	Improving cardiac function and alleviating fibrosis	MiR-22/methyl CpG binding protein 2	(115)
4	Kidney	Tubular epithelial cells	Attenuating tubular epithelial-myofibroblast transdifferentiation of renal tubular epithelial cells	MiR-335-5p/ADAM19	(116)
5	Colon	Intestinal epithelial cells	Inhibition of EMT	MiR-200b/ZEB1, ZEB2	(117)
		Macrophage	Reduction of inflammatory cytokines	MiR-146a/TRAF6, IRAK1	(118)

MSC-EVs, mesenchymal stem cell-derived extracellular vesicles; MSC, mesenchymal stem cell; EMT, epithelial-to-mesenchymal transition.

#### 3.3.1 Antifibrotic function of MSC-EVs

MSC-EVs have been confirmed to be beneficial for SSc due to their antifibrotic effects. Rozier et al. evaluated the antifibrotic function of ASCs and their EVs by co-culturing them with TGF-β1-induced myofibroblasts (65). However, the underlying therapeutic mechanisms remain unclear. Several studies have investigated the potential antifibrotic properties of MSC-EVs in bleomycin-induced mouse models of scleroderma (64, 134). These effects were exhibited in the amelioration of the following aspects. MSC-EVs can suppress myofibroblast differentiation and inhibit the expression of collagen types I and III in a skin-defect mouse model (111). Studies on the molecular markers of fibrosis and remodeling have shown that MSC-EVs might diminish scar formation by decreasing the ratios between collagen types I and III in a diabetic mouse model (138). In addition, ASC-EVs decrease scar formation *via* modulating the ratios of type I and III collagen, TGF-β1 and TGF-β3, matrix metalloproteinase-3 and tissue inhibitor of metalloproteinases 1, and abnormal activation of fibroblasts *in vivo* (139). These effects have been noted in many fibrotic diseases (140).

#### 3.3.2 Antifibrotic molecular mechanisms of MSC-EVs

Many studies have confirmed that MSC-EVs, typified by Exos, can act as antifibrotic agents through various mechanisms of action in the treatment of fibrotic diseases. Among the signaling pathways involved, TGF-β and Wnt signaling are regarded as the core pathways involved in fibrosis in SSc (141, 142). In addition, the effects of Exos on reducing fibrotic markers through the TGF pathway have also been confirmed in SSc (64). The phosphorylation levels of α-SMA, Smad2/3, and collagen I and III in fibroblasts treated with Exos have been found to be markedly decreased. Furthermore, MSC-derived Exos have been found to suppress the transition of dermal fibroblasts to myofibroblasts by inhibiting the NF-κB, Hedgehog, Wnt/β-catenin, PI3K/Akt, Erk1/2, and TGF-β1/Smad2/3 signaling pathways (140). Moreover, it has been shown that EV alleviates fibrosis by carrying cargo, such as RNA, through activation of the aforementioned signaling pathways. Results of high-throughput RNA sequencing and functional analysis of MSC-EVs suggest that a group of specific microRNAs can prevent excessive myofibroblast revitalization by blocking the TGF-β/Smad2 signaling pathways *in vivo* (111). MiR-29b-3p, carried by BM-MSC-EVs in bilayered thiolated alginate/PEG diacrylate hydrogels, inhibits the proliferation and migration of ECs and fibroblasts by curbing the PI3K/Akt, Erk1/2, and TGF-β1/Smad3 signaling pathways in a full-thickness skin defect model of rats and rabbit ears, thereby allowing for scar-free wound healing (110). In a study on BMSC-EV-based treatment, the gene activity in the Wnt signaling pathway that was hyperactivated by bleomycin stimulation was significantly lowered or related to ECM–receptor interactions and the cell cycle (68).

Several investigations have explored the role of MSC-EV-carried miRNAs in fibrosis. In SSc, miR-29a-3p from MSCs and ASCs has been confirmed to be beneficial for fibrosis in HOCl-induced mice (69). In other fibrotic diseases, a group of uMSC-EVs composed of miR-21, miR-23a, miR-imno125b, and miR-145, whose expression is changed, was thought to be related to the modulation of pro-fibrotic genes (110, 111). Among the various features of fibrosis, MSC-EVs also display efficacy in the inhibition of fibroblast proliferation. MiR-29b-3p and miR-186, both secreted by BMMSC-EVs, have been reported to downregulate FZD6, SOX4, and DKK1 expression to inhibit fibroblast activation and proliferation in idiopathic pulmonary fibrosis, respectively (108, 109). Likewise, elevated collagen levels are one of the main factors contributing to ECM deposition in fibrosis. MiR-212-5p, miR-133b (143), miR-192-5p (144), miR-196b-5p (67), and let-7a (145) have been found to reduce the levels of type I and/or type III collagens, and miR-212-5p downstream may inhibit the NLRC5/VEGF/TGF-β1/SMAD axis, while miR-192-5p targets IL-17RA. Similarly, fibrosis and remodeling factors are targets of miRNAs from MSC-EVs. EVs from MSCs and ASCs exhibit a mechanism of action that comprises the secretion of miR-29a-3p, which could diminish the levels of several profibrotic, remodeling, and anti-apoptotic factors and methylases in HOCl-induced mice (69). In the aforementioned studies, MSC-EVs further exerted a positive effect on fibrosis. Despite the progress that has been made in recent studies, ameliorating fibrosis with MSC-EVs remains in the preclinical stage.

## 4 Conclusions and prospects

It is possible that MSC-EVs could ameliorate SSc and the pathological changes in vasculopathy, immune dysfunction, and fibrosis by regulating key factors and signaling pathways. Despite the positive effects of MSC-EVs in SSc, further extensive clinical studies are required to establish the applicability of EVs in treating SSc. A focus area is modification and engineering, which could enhance the therapeutic effects of MSCs-EVs. Future exploration of strategies, including chemical stimulation from MSCs, MSC genetic modification, and physical variables of MSCs, could generate more effective MSCs-EVs for supporting the desired outcomes. Undoubtedly, enhancing the effectiveness of MSC-EV treatment will be an important trend in future clinical applications.

## Author contributions

NS participated in the investigation. JJ and PL reviewed and supervised the manuscript. All authors contributed to the paper and approved the submitted draft. All authors contributed to the article and approved the submitted version.

## Glossary

**Table:**

AD	autoimmune disease
AF-MSC	amniotic fluid-derived mesenchymal stem cell
APC	antigen-presenting cell
ASC	adipose tissue-derived mesenchymal stem cell
BAFF	B-cell activating factor
BM-MSC	bone marrow-derived mesenchymal stem cell
Breg	B-regulatory cell
CTGF	connective tissue growth factor
CCR7	C–C chemokine receptor type 7
DFU	diabetic foot ulcer
EC	endothelial cell
ECM	extracellular matrix
EMT	epithelial-to-mesenchymal transition
EV	extracellular vesicle
Exos	Exosomes
FGF	fibroblast growth factor
HLA	human leukocyte antigen
HMEC	human microvascular endothelial cell
HOCl	hypochlorous acid
HUVEC	human umbilical vein endothelial cell
IFN	interferon
IL	interleukin
lncRNA	long non-coding RNA
IPF	idiopathic pulmonary fibrosis
MAPK	mitogen-activated protein kinase
MenSC	menstrual fluid-derived mesenchymal stem cell
MI	myocardial infarction
miRNA	microRNA
MSC	mesenchymal stem cell
MSC-EV	mesenchymal stem cell-derived extracellular vesicle
NK	natural killer
pDC	plasmacytoid dendritic cell
PDGF	platelet-derived growth factor
SSc	systemic sclerosis
SSc-Fb	fibroblasts from patients with systemic sclerosis
SSc-ILD	SSc-associated interstitial lung disease
Tfh	T-follicular helper cell
TGF	transforming germinal factor
Tβ-Fb	TGF-β1-induced myofibroblasts
TLR	Toll-like receptor
TNF	tumor necrosis factor
Treg	T-regulatory
UC-MSC	umbilical cord-derived mesenchymal stem cell
VEGF	vascular endothelial-derived growth factor
VSMC	vascular smooth muscle cell
