# Supplementary material for: Potential angiogenic, immunomodulatory, and antifibrotic effects of mesenchymal stem cell-derived extracellular vesicles in systemic sclerosis
Source: Front Immunol. 2023 May 12;14:1125257. doi: 10.3389/fimmu.2023.1125257 (PMC10213547; doi:10.3389/fimmu.2023.1125257)
Supplement: Supplementary file 1 [file Table_1.docx]

Supplementary Material

Potential Angiogenic, Immunomodulatory, and Antifibrotic Effects of Mesenchymal Stem Cell-Derived Extracellular Vesicles in Systemic Sclerosis

Kelin Zhao1†, Chenfei Kong2†, Naixu Shi 3, Jinlan Jiang2* and Ping Li1*

^1^ Department of Rheumatology and Immunology, China-Japan Union Hospital, Jilin University, Chang-chun, China.

^2^ Scientific Research Center, China-Japan Union Hospital, Jilin University, Changchun, China.

^3^ Department of Stomatology, China-Japan Union Hospital, Jilin University, Changchun, China.

*** Correspondence:**

Jinlan Jiang

jiangjinlan@jlu.edu.cn

Ping Li

li_ping@jlu.edu.cn

†These authors contributed equally to this work and share first authorship

# Supplementary Table

| **Table 1.** Summary of disease manifestations of SSc models | | | | | | | |
| --- | --- | --- | --- | --- | --- | --- | --- |
|  | **Vitro/Vivo** | **Classification** | **Models** | **Animals** | **Methods** | **Main Characteristics** | **Reference** |
| 1 | vivo | Chemically inducing | Bleomycin | Balb/C mice or C57BL/6 mice | Subcutaneous or intravenous injection | Inflammation: T cells, monocytes/macrophages, mast cell and antibodies | (1) |
|  |  |  |  |  |  | Fibrosis: skin and lung |  |
| 2 |  |  | HOCl | BALB/c, C57BL6, DBA/1 or NZB mice | Subcutaneous or intravenous injection | Vasculopathy: sVCAM-1 and sE-selectin | (2) |
|  |  |  |  |  |  | Inflammation: B-cell, CD4þ T-cell and antibodies |  |
|  |  |  |  |  |  | Fibrosis: skin |  |
| 3 |  |  | Sclerodermatous-GVHDs | BALB/c mice | Bone marrow allografts containing only lymphocytes | Inflammation: CD4+ T cells | (3) |
|  |  |  |  |  |  | Fibrosis:IL-2, IL-10, and TGFβ |  |
| 4 |  |  | Topoisomerase-1 | C57BL/6 mice | Immunized with recombinant topo I protein emulsified in Freund’s complete adjuvant | Inflammation:IL-6,TGF-β1， IL-17 and IL-10 | (4) |
| 5 |  |  |  |  |  | Fibrosis:skin and lung |  |
| 6 |  |  | Skin-humanized mice | Severe combined immunodeficiency (SCID) gene mice | Transplantation of fibroblasts from SSc patients to the back of SCID mice. | Fibrosis:collagen deposition and fibroblast activation | (5) |
| 7 |  |  | Type V collagen | C57BL/6 mice | Subcutaneously immunized with Col V mulsified in complete Freund adjuvant | Vasculopathy: endothelial cell and VEGF, endothelin-1, and caspase-3 | (6) |
|  |  |  |  |  |  | Fibrosis:COL1A1, COL1A2, COL3A1, COL5A1, and COL5A2 in skin |  |
| 1 |  | Genetic manipulation | Tsk-2 | Tsk2+/- mice | Mice with Tsk2+/- heterozygotes | Fibrosis:type III collagen in skin | (7) |
| 2 |  |  | Fli-1 ko | Fli-1-/- mice | Injection of bleomycin after silencing of Fli-1 gene | Vasculopathy:change the molecules involved in maintaining vascular homeostasis | (8) |
|  |  |  |  |  |  | Fibrosis: TGFβ in dermal fibroblasts | (9) |
| 3 |  |  | Fli1-KLF5-KO | Klf5+/-Fli1+/- mice | Mice with heterozygous deficiency of both Klf5 and Fli1 | Vasculopathy:obliterative vasculopathy and lung fibrosis | (10) |
|  |  |  |  |  |  | Inflammation:B cell activation, and autoantibody production |  |
|  |  |  |  |  |  | Fibrosis:skin and lung |  |
| 4 |  |  | uPAR-KO | uPAR−/− mice | Silencing of the uPAR gene | Vasculopathy: perivascular cell and endothelial cell | (11) |
|  |  |  |  |  |  | Fibrosis:skin |  |
| 5 |  |  | Sirt3-KO | Mice with Sirt3 and mice with the infusion of Ang-II | Crossing loxP-stop-LoxP-SIRT3.Flag-transgenic mice with mice | Fibrosis:skin,lung, heart and kidney | (12) |
| 6 |  |  | Fra-2 Tg | Fra-2 Transgenic Mice | Transgenic mice expressing Fra-2 under the control of H2Kb promoter | Vasculopathy:dermal capillary rarefaction and obliterative vasculopathy | (13) |
|  |  |  |  |  |  | Fibrosis:spontaneous skin fibrosis |  |
| 7 |  |  | Fbn-1 | Fbn-1-deficient mice | Homologous recombinant Fbn1D1545E/+ and Fbn1W1572C/+ mice | Inflammation:dendritic cell, macrophage activation and antibodies | (14) |
|  |  |  |  |  |  | Fibrosis:stiff skin syndrome and loss of white adipose tissue |  |
| 8 |  |  | TβRI | C57BL6/J mice | Injection of adenovirus expressing a constitutively active form of TGF-β | Fibrosis:local fibrosis | (15) |
| 9 |  |  | VEGF | VEGF+/- tg mice | Mice with homozygous VEGF+/+ or heterozygous VEGF+/- | Vasculopathy: an inverse gene-dosing effect on the efficacy of angiogenesis | (16) |
|  |  |  |  |  |  | Fibrosis:skin |  |
| 10 |  |  | PTEN KO | loxP-Ccn2 mice | loxP-Ccn2 mice and mice expressing tamoxifen-dependent Cre recombinase | Fibrosis:skin and lung | (17) |
| 11 |  |  | Type VI collagen | A double transgenic mouse strain employing a tetracycline-inducible system | Transgenic mice overexpressing endotrophin | Fibrosis:selective expansion of white adipose tissue and modulation fibrogenesis in different anatomic sites, | (18) |
| 1 | vitro | Chemically inducing | TGF-β |  | Addition of TGFβ1 to human fibroblasts after starvation | Fibrosis: transformation of fibroblasts into myofibroblasts | (19) |

1. Blyszczuk P, Kozlova A, Guo Z, Kania G, Distler O. Experimental Mouse Model of Bleomycin-Induced Skin Fibrosis. *Curr Protoc Immunol* (2019) 126(1):e88. Epub 2019/09/05. doi: 10.1002/cpim.88.

2. Meng M, Tan J, Chen W, Du Q, Xie B, Wang N, et al. The Fibrosis and Immunological Features of Hypochlorous Acid Induced Mouse Model of Systemic Sclerosis. *Front Immunol* (2019) 10:1861. Epub 2019/09/05. doi: 10.3389/fimmu.2019.01861.

3. Schroeder MA, DiPersio JF. Mouse Models of Graft-Versus-Host Disease: Advances and Limitations. *Dis Model Mech* (2011) 4(3):318-33. Epub 2011/05/12. doi: 10.1242/dmm.006668.

4. Yoshizaki A, Yanaba K, Ogawa A, Asano Y, Kadono T, Sato S. Immunization with DNA Topoisomerase I and Freund's Complete Adjuvant Induces Skin and Lung Fibrosis and Autoimmunity Via Interleukin-6 Signaling. *Arthritis Rheum* (2011) 63(11):3575-85. Epub 2011/07/28. doi: 10.1002/art.30539.

5. Luchetti MM, Moroncini G, Jose Escamez M, Svegliati Baroni S, Spadoni T, Grieco A, et al. Induction of Scleroderma Fibrosis in Skin-Humanized Mice by Administration of Anti-Platelet-Derived Growth Factor Receptor Agonistic Autoantibodies. *Arthritis Rheumatol* (2016) 68(9):2263-73. Epub 2016/04/26. doi: 10.1002/art.39728.

6. Teodoro WR, de Jesus Queiroz ZA, Dos Santos LA, Catanozi S, Dos Santos Filho A, Bueno C, et al. Proposition of a Novel Animal Model of Systemic Sclerosis Induced by Type V Collagen in C57bl/6 Mice That Reproduces Fibrosis, Vasculopathy and Autoimmunity. *Arthritis Res Ther* (2019) 21(1):278. Epub 2019/12/13. doi: 10.1186/s13075-019-2052-2.

7. Long KB, Li Z, Burgwin CM, Choe SG, Martyanov V, Sassi-Gaha S, et al. The Tsk2/+ Mouse Fibrotic Phenotype Is Due to a Gain-of-Function Mutation in the Piiinp Segment of the Col3a1 Gene. *J Invest Dermatol* (2015) 135(3):718-27. Epub 2014/10/21. doi: 10.1038/jid.2014.455.

8. Saigusa R, Asano Y, Yamashita T, Taniguchi T, Takahashi T, Ichimura Y, et al. Fli1 Deficiency Contributes to the Downregulation of Endothelial Protein C Receptor in Systemic Sclerosis: A Possible Role in Prothrombotic Conditions. *Br J Dermatol* (2016) 174(2):338-47. Epub 2015/09/25. doi: 10.1111/bjd.14183.

9. Taniguchi T, Asano Y, Akamata K, Noda S, Takahashi T, Ichimura Y, et al. Fibrosis, Vascular Activation, and Immune Abnormalities Resembling Systemic Sclerosis in Bleomycin-Treated Fli-1-Haploinsufficient Mice. *Arthritis Rheumatol* (2015) 67(2):517-26. Epub 2014/11/12. doi: 10.1002/art.38948.

10. Noda S, Asano Y, Nishimura S, Taniguchi T, Fujiu K, Manabe I, et al. Simultaneous Downregulation of Klf5 and Fli1 Is a Key Feature Underlying Systemic Sclerosis. *Nat Commun* (2014) 5:5797. Epub 2014/12/17. doi: 10.1038/ncomms6797.

11. Manetti M, Rosa I, Milia AF, Guiducci S, Carmeliet P, Ibba-Manneschi L, et al. Inactivation of Urokinase-Type Plasminogen Activator Receptor (Upar) Gene Induces Dermal and Pulmonary Fibrosis and Peripheral Microvasculopathy in Mice: A New Model of Experimental Scleroderma? *Ann Rheum Dis* (2014) 73(9):1700-9. Epub 2013/07/16. doi: 10.1136/annrheumdis-2013-203706.

12. Sundaresan NR, Bindu S, Pillai VB, Samant S, Pan Y, Huang JY, et al. Sirt3 Blocks Aging-Associated Tissue Fibrosis in Mice by Deacetylating and Activating Glycogen Synthase Kinase 3beta. *Mol Cell Biol* (2015) 36(5):678-92. Epub 2015/12/17. doi: 10.1128/MCB.00586-15.

13. Eferl R, Hasselblatt P, Rath M, Popper H, Zenz R, Komnenovic V, et al. Development of Pulmonary Fibrosis through a Pathway Involving the Transcription Factor Fra-2/Ap-1. *Proc Natl Acad Sci U S A* (2008) 105(30):10525-30. Epub 2008/07/22. doi: 10.1073/pnas.0801414105.

14. Gerber EE, Gallo EM, Fontana SC, Davis EC, Wigley FM, Huso DL, et al. Integrin-Modulating Therapy Prevents Fibrosis and Autoimmunity in Mouse Models of Scleroderma. *Nature* (2013) 503(7474):126-30. Epub 2013/10/11. doi: 10.1038/nature12614.

15. Palumbo-Zerr K, Zerr P, Distler A, Fliehr J, Mancuso R, Huang J, et al. Orphan Nuclear Receptor Nr4a1 Regulates Transforming Growth Factor-Beta Signaling and Fibrosis. *Nat Med* (2015) 21(2):150-8. Epub 2015/01/13. doi: 10.1038/nm.3777.

16. Maurer B, Distler A, Suliman YA, Gay RE, Michel BA, Gay S, et al. Vascular Endothelial Growth Factor Aggravates Fibrosis and Vasculopathy in Experimental Models of Systemic Sclerosis. *Ann Rheum Dis* (2014) 73(10):1880-7. Epub 2013/08/07. doi: 10.1136/annrheumdis-2013-203535.

17. Parapuram SK, Shi-wen X, Elliott C, Welch ID, Jones H, Baron M, et al. Loss of Pten Expression by Dermal Fibroblasts Causes Skin Fibrosis. *J Invest Dermatol* (2011) 131(10):1996-2003. Epub 2011/06/10. doi: 10.1038/jid.2011.156.

18. Sun K, Park J, Gupta OT, Holland WL, Auerbach P, Zhang N, et al. Endotrophin Triggers Adipose Tissue Fibrosis and Metabolic Dysfunction. *Nat Commun* (2014) 5:3485. Epub 2014/03/22. doi: 10.1038/ncomms4485.

19. Rozier P, Maumus M, Bony C, Maria ATJ, Sabatier F, Jorgensen C, et al. Extracellular Vesicles Are More Potent Than Adipose Mesenchymal Stromal Cells to Exert an Anti-Fibrotic Effect in an in Vitro Model of Systemic Sclerosis. *Int J Mol Sci* (2021) 22(13). Epub 2021/07/03. doi: 10.3390/ijms22136837.
